# Chemistry and Biology of Bengamides and Bengazoles, Bioactive Natural Products from *Jaspis* Sponges

**DOI:** 10.3390/md12031580

**Published:** 2014-03-18

**Authors:** Cristina García-Ruiz, Francisco Sarabia

**Affiliations:** Department of Organic Chemistry, Faculty of Sciences, University of Málaga, Campus de Teatinos s/n., Málaga 29071, Spain; E-Mail: crisgarcia@uma.es

**Keywords:** bengamides, bengazoles, antitumor, analogues, total synthesis

## Abstract

Sponges corresponding to the *Jaspidae* family have proved to be a prolific source of bioactive natural products. Among these, the bengamides and the bengazoles stand out by virtue of their unprecedented molecular architectures and impressive biological profiles, including antitumor, antibiotic and anthelmintic properties. As a consequence, intense research activity has been devoted to these compounds from both chemical and biological standpoints. This review describes in detail the research into these classes of natural products and the benefits they offer in chemistry and biology.

## 1. Introduction

**Figure 1:**
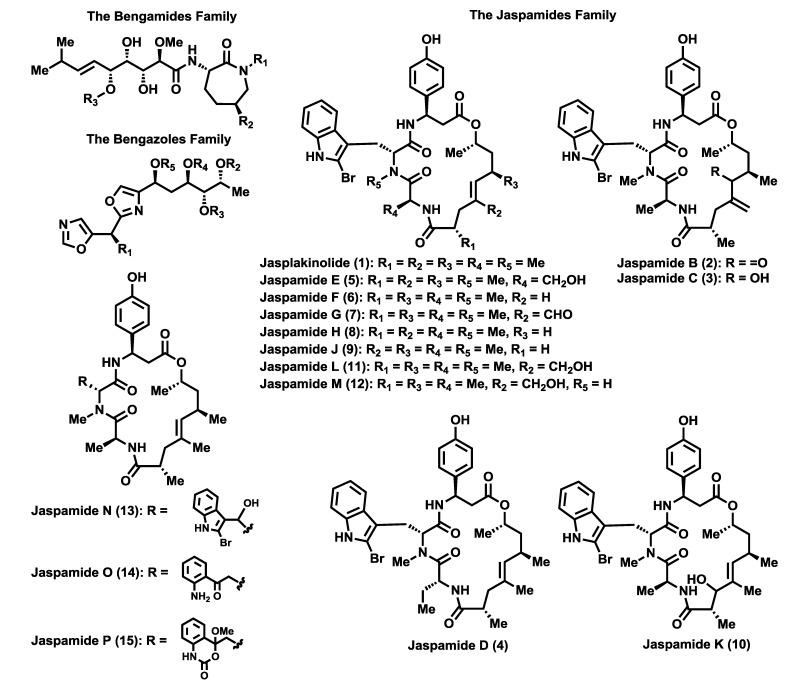
Natural products from *Jaspidae* Sponges: bengamides, bengazoles and jaspamides.

The sponges corresponding to the *Jaspidae* family represent a valuable source of interesting natural products, some of which display impressive biological profiles such as antitumoral, anthelmintic and antibiotic properties. This class of sponges has been mainly collected in the Indian Ocean and has provided a wide array of natural products in terms of structural diversity and biological properties, although there are three classes of natural compounds that stand out over the rest, the bengamides [1], the bengazoles [2] and the jaspamides [3] (Figure 1) due to their unique molecular architectures and biological activities. The bengamides and bengazoles, whose first members were discovered in 1986, represent unique natural products and will be covered in detail in this review. On the other hand, the cyclodepsipeptide jaspamide A or jasplakinolide (**1**), independently isolated by the Crews and the Ireland research groups in 1986 [3], possesses an impressive antitumor profile that also has elicited intense research activity [4]. Its isolation from *Jaspis johnstoni* [5] was followed by the discovery of new members, the jaspamides B–P (**2**–**15**) [6,7,8,9], from other sponges belonging to the *Jaspis* family and with similar striking antitumor properties.

**Figure 2:**
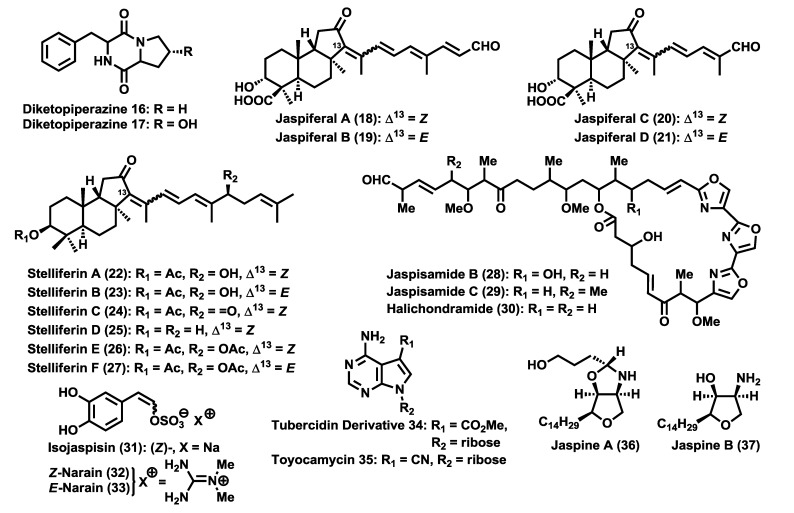
Natural products from *Jaspidae* sponges: other secondary metabolites.

In addition to aforementioned families of natural products, many other bioactive compounds have been isolated from these sponges such as diketopiperazines (**16**, **17**) [10], which possess modest cytotoxic activities, the jaspiferals (**18**–**21**) [11] and stelliferins (**22**–**27**) [12], isomalabaricane triterpenoids with cytotoxic, antifungal and antibacterial activities [13,14], jaspisamides (**28**, **29**) and halichondramide (**30**) [15], oxazole-containing macrolides with very potent antitumoral activities, the styryl sulfates isojaspisin (**31**) [16] and narains (**32**, **33**) [17], that inhibit hatching of sea urchin embryos, the nucleosides **34** or **35** [18], with anticandidal activities and modest cytotoxicity against leukemia and the jaspines (**36**, **37**) [19] (Figure 2), with remarkable cytotoxic activities and as pro-inflammatory agonists.

Table 1 summarizes the isolation of all these secondary metabolites, indicating the taxonomic identification of the natural source and the location where these sponges were collected. Some of the aforementioned natural products were similarly isolated from other species of marine sponges (information contained in Table 1).

The present review focuses on the chemistry and biology of the bengamides and the bengazoles, which, due to their interesting biological properties, has prompted a flurry of research activity in the chemical, biological and biomedical fields in the last years. Taking into account that all this research activity has not been reviewed yet, we consider that the current article may reflect the state of the art on this issue by describing all the achievements in the chemical and biological fields regarding these natural products. Additionally, we provide a perspective of the opportunities that these compounds may offer in the future, as promising new bioactive compounds for the treatment of diseases.

**Table 1:** Metabolites from *Jaspis* sponges.

Taxonomic Identification	Collection Site	Isolated Compounds
*Jaspis* cf. *coriacea*	Fiji Island, Solomon Island, Papua, New Guinea, Indonesia	Jasplakinolide [3] ^a^, Bengamides A–F, Isobengamide E, Bengazoles A and B ^b^, Diketopiperazine cyclo (l-*trans* (4-hydroxyprolinyl)-l-Phe) and *N*-acetyl-l-phynylalanine [10], Bengamides M–R [20] ^c^
*Jaspis carteri*	New Caledonia	Bengamide A–B, G–J, K [21]
*Jaspis digonoxea*	Indo-Pacific region, South Africa	Digonazole [22]
*Jaspis stellifera*	Okinawa	Jaspiferals A–G [11], Stelliferins A–F [12] ^d^
*Jaspis splendans*	Vanuatu Islands	Jaspamides B–C [6] ^a^, Jaspamides B–G [7], Jaspamides H–L [3] ^a^, Jaspamides M–P [9] ^a^
*Jaspis* cf. *johnstoni*	Fiji Island	Jaspamide [5] ^a^, Toyokamycin, 5-(Methoxycarbonyl)tubercidin [18]
*Jaspis* sp.	Northern Great Barrier Reef	Bengazoles A–G and B1 [23] ^b^
*Jaspis* sp*.*	Okinawa	Jaspisamides A–C [15]
*Jaspis* sp.	Izu Peninsula	3,4-Dihydroxystyrene *E* and *Z*; (*E*) and (*Z*)-narains [17]
*Jaspis* sp.	Okino-shima Island	Isojaspisin [24]
*Jaspis* sp.	Indo-Pacific region	Bengazoles A–B, C–G [25] ^b^
*Jaspis* sp.	Djibouti (Red Sea, Gulf of Aden)	Jaspines A–B [19] ^e^

^a^ Jaspamides have been also isolated from the marine sponge *Cymbastela* sp. [26]; ^b^ Bengazoles Z, C4 and C6 were additionally isolated from the sponge *Stelleta* sp. [27]; ^c^ Bengamides E, E′ and F′ were found in *Myxococcus virescens* [28], bengamides A, F, N and Y from the sponge *Stelleta* sp. [27] and bengamide L from *Pachastrissa* sp. [29]; ^d^ Stelliferins J–N were also isolated from the Okinawan marine sponge *Rhabdastrella* cf. *globostellata* [30], *Stellata globostellata* [31] and *Geodia globostellifera* [32]. ^e^ Jaspines were also described from *Pachastrissa* sp. [33].

## 2. Chemistry and Biology of the Bengamides

### 2.1. Discovery, Structural Determination and Biological Properties

**Figure 3:**
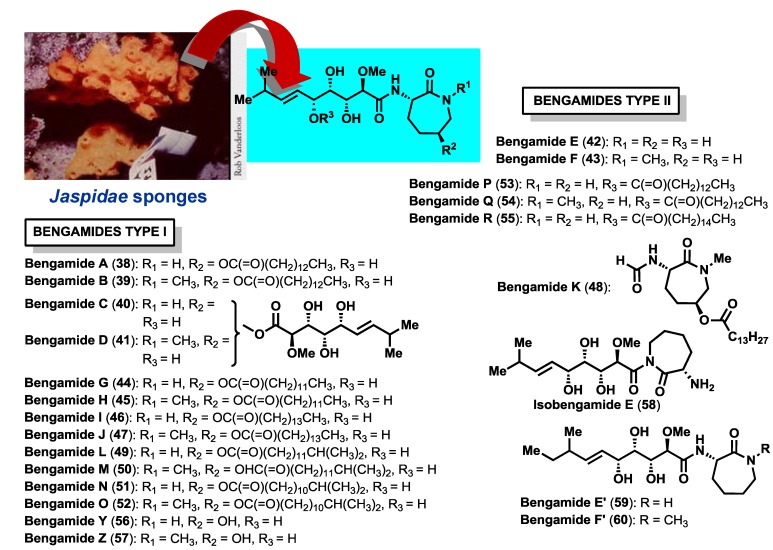
Molecular structures of bengamides.

The bengamide family is comprised of a wide number of members (Figure 3), with the bengamides A–F the first to be discovered and isolated between 1986 [1] and 1989 [10] by the research group of Professor Crews (University of California, Santa Cruz, CA, USA) from an undescribed specimen of an orange sponge belonging to the *Jaspidae* family (family Choristida, order Astrophorida) that was collected in Benga Lagoon (Fiji Islands). The crude extract obtained from these sponges exhibited an impressive cytotoxicity profile against larynx epithelial carcinoma (1.0 μg/mL) and striking anthelmintic and antibacterial activities against the nematode *Nippostrongylus braziliensis* and *Streptococcus pyrogenes* [1]. An extensive spectroscopic analysis led Crews and coworkers to establish the structures for the main components of this crude corresponding to bengamides A (**38**) and B (**39**). A third component, bengamide C (**40**), was also identified in the crude extract, however, its structure determination was not possible at that time because it was not obtained in pure form. After two years the research group of Professor Crews was able to recollect huge amounts of this sponge and isolate new compounds related to the first bengamides, characterized as bengamides C–F (**40**–**43**), together with the related isomeric product isobengamide E (**58**) and various oxazole derivatives, named bengazoles, which will be described in detail later in the review [10,34]. After this discovery, the extracts of the sponge *Jaspis carteri*, collected in New Caledonia, showed a remarkable anticandidal activity and, after an expeditious purification process and structural analysis, the research group led by D’Auria isolated and identified bengamides A and B, together with a series of new members, including bengamides G–J (**44**–**47**) and a truncated derivative, bengamide K (**48**) [21]. Later, in 1999, Groweiss [2] and co-workers isolated from *Jaspis* sp. two new members of the bengamides; bengamide Y (**56**), which lacks the fatty acid moiety and the *N*-methyl group at position 15, and bengamide Z (**57**), the nonacylated derivative of bengamide B. In the same year, Letourneux *et al.* [29] isolated from sponge *Pachastrissa* sp. (family Calthropellidae, order Astrophorida) bengamide L (**49**), together with novel members of the bengazoles. More recently, in 2001, more than 15 years after the discovery of the first bengamides, Crews *et al.* [20] described six new bengamides, bengamides M–R (**50**–**55**) as well as the known bengamides A (**38**), B (**39**), E (**42**), F (**43**), G (**44**), H (**45**), I (**46**), L (**49**), Y (**56**) and Z (**57**), from a collection of *Jaspis* cf. *coriacea*. The last contribution to this numerous family of natural products was generated also by Crews [28] in 2012, who identified the bacteria *Myxococcus virescens* as a new source of bengamide E (**42**), and two new congeners, bengamides E′ (**59**) and F′ (**60**), isolated as an inseparable mixture of diastereomers, thus being the second example, in the peer-reviewed literature, of a sponge-derived natural product reported from a cultured microbial source (the first one being Makaluvamine A, isolated from the sponge *Zyzzya fuliginosa* [35] and myxomycetes *Didymium bahiense* [36]). It is also interesting to point out that bengamide E′ was initially synthesized in the laboratory prior to its isolation from a natural source [37]. Currently, 23 members of the bengamide family have been described and their molecular structures determined by spectroscopic methods.

The bengamide structures contain a unique skeleton with a C-10 side chain possessing four contiguous hydroxyl groups as well as an *E*-olefin, that links to an aminocaprolactam moiety. According to the caprolactam ring, the bengamides have been classified into two structural classes: Type I that contains a hydroxylysine-derived caprolactam, bearing or not a lipidic chain (bengamides A–D, G–J, L–O, Y, Z) and Type II that contains a lysine-derived caprolactam (bengamides E, F, E′, F′, P–R).

**Scheme 1:**
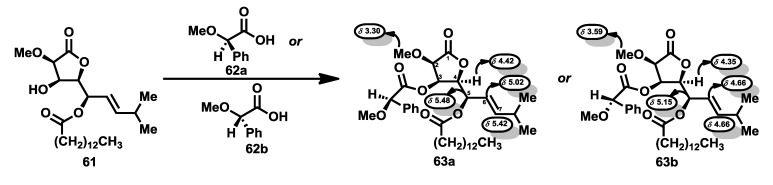
Chemical studies for structure determination.

Structure determination of the bengamides was carried out by Crews *et al.* [38], as mentioned previously. Thus, the establishment of the absolute stereochemistry was possible by means of spectroscopic studies of the ^1^H-NMR of the (*R*)- and (*S*)-mandelate esters of lactone **61**, a product obtained during the isolation process. Thus, these esters, compounds **63a** and **63b**, prepared by reaction of **61** with the O-methyl mandelic acids **62a** and **62b** respectively, showed significant differences in their respective ^1^H NMR spectra, particularly upfield shifts of H-4, H-5, H-6 and H-7 and downfield shifts for H-2 and the methoxyl group for the *S* derivative **63b** in comparison to the *R* derivative **63a**. The validity of the O-methylmandelate ester method [39] was checked with the preparation of the corresponding esters of (−)-menthol, in which similar effects were observed in the chemical shifts of key protons (Scheme 1). Subsequent total syntheses of bengamides A [40], B [41] and E [42], as will be discussed in detail in Section 2.3, confirmed the absolute configurations for the C10 side chain and for the hydroxylysine-derived caprolactam.

**Figure 4:**
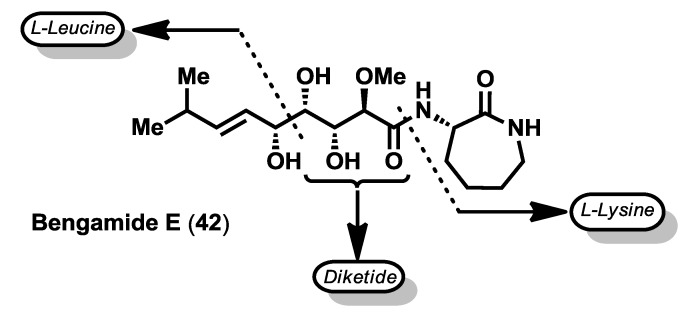
Proposed biosynthetic origin of the bengamides.

Interestingly, the biosynthetic origin of the bengamides seems to be the result of a symbiotic interaction between this class of sponges and bacteria, as revealed from their structures as the end-chain isopropyl group, characteristic of bacterial fatty acids [43,44]. Thus, the structure of the bengamides may arise from the linkage of a diketide with a six-carbon unit derived from l-leucine to form the C10 side chain, which couples with cyclized l-lysine (Figure 4) [10].

As indicated earlier, preliminary biological studies of the bengamides revealed *in vitro* cytotoxicity towards larynx epithelial carcinoma at 1.0 µg/mL, together with activity against the bacteria *Streptococcus pyrogenes* and the nematode *Nippostrongylus braziliensis* [1]. However, the bengamides proved inactive in assays against *Candida albicans* and *Saccharomyces cerevisiae*, in contrast to the bengazoles, which displayed potent antifungal activity [29], as will be discussed later. Subsequent biological evaluations demonstrated the potent antiproliferative activities displayed by some members of the bengamides against different tumor cell lines, with IC_50_ values ranging from 10 to 100 nM [20]. Additionally, Crews *et al.* [28] recently discovered that the bengamides behave as immune modulating agents owing to their inhibition of NF-κB (nuclear factor kappa B) without accompanying cytotoxicity to RAW 264.7 macrophage immune cells, with the bengamides A (**38**) and B (**39**) as the most potent inhibitors. These studies suggest that the bengamides may serve as therapeutic leads for the treatment of diseases involving inflammation.

### 2.2. Antitumor Properties and Mechanism of Action

Intense cytotoxic studies of the antitumor activities of the bengamides, together with the results provided by the NCI-DTP database, has resulted in their identification as promising new anticancer agents [20]. Thus, IC_50_ values for natural bengamides A (**38**), B (**39**), E (**42**), F (**43**), M (**50**), O (**52**), P (**53**) and Z (**57**) were determined against MDA-MB-435 human mammary carcinoma with the best anti-proliferative *in vitro* activities observed for bengamides having a fatty acid attached to the caprolactam ring (cases of bengamides A, B, M and O), which were >100-fold more potent than their nonlactam ester-bearing counterparts (bengamides E, F, P and Z) (Table 2). However, despite the importance of a lipohilic ester on the caprolactam moiety for *in vitro* potency, *in vivo* studies revealed small differences in antitumor activity between all the bengamides, likely due to their poor water solubilities. On the other hand, it was demonstrated that bengamide B (**39**) was converted to bengamide Z (**57**) intracellularly, which suggests that this compound (**57**) is actually responsible for the antiproliferative effects [45]. Thus, the difference of activities observed *in vitro* for bengamides B and Z likely arises from the poor cellular uptake of **57**. Among all the natural bengamides, bengamide B (**39**) displayed a unique profile in the NCI 60 cell line panel compared to standard antitumor agents, revealing arrest at both G1 and G2/M phases of the cell cycle by FACS (Fluorescence-activated cell sorter) analyses of transformed and non-transformed cells, respectively. Additional biological experiments led to the conclusion that the G1 arrest occurred at the G1/S restriction point and that the cells arrested in the G2/M phase of the cell cycle were not inhibited during mitosis but rather during cytokinesis [20,46,47]. All these biological data suggested that the cytotoxicity exhibited by the bengamides was due to inhibition of a novel target [48].

**Table 2:** *In vitro* anti-proliferative activity of selected natural bengamides (IC_50_ [µM]) on MDA-MB-435 human mammary carcinoma cells.

Bengamide	MDA-MB-435
A (**38**)	0.001 ± 0.0006
B (**39**)	0.012 ± 0.003
M (**50**)	0.0101 ± 0.0021
O (**52**)	0.00029 ± 0.0005
Z (**57**)	2.9 ± 1.5
E (**42**)	3.3 ± 1.2
F (**43**)	2.9 ± 2.9
P (**53**)	1.2 ± 7.9

**Figure 5:**
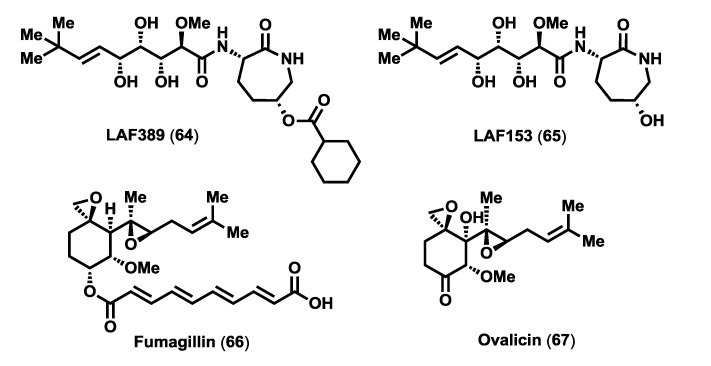
Inhibitors of MetAPs: bengamide analogues LAF389 and LAF153, Fumagillin and Ovalicin.

In 2003, Towbin *et al.* [48], in an effort to elucidate the mechanism of action of the bengamides, undertook extensive biological studies that allowed them to exclude many relevant biological targets involved in cancer, such as DNA, tubulins, actin, topoisomerases or proteases. On the other hand, proteomic studies performed on H1299 cells [48] detected an alteration in a subset of proteins, the 14-3-3 protein isoforms, after treatment *in vitro* with LAF389 (**64**) (Figure 5), a more soluble synthetic analogue of bengamides, with striking inhibitory effects on tumor growth *in vitro* and *in vivo* [49]. As a consequence of these results, a more detailed study focused on the 14-3-3 protein family (cytosolic adaptor proteins that modulate intracellular signaling, cell cycle control, transcriptional control and apoptosis [50]) revealed a retention of the initiator methionine in the isoform 14-3-3 when treated with the bengamides, and consequently allowed to determine the direct targets of bengamides as both human methionine aminopeptidases (MetAP) isoforms, MetAP1 and MetAP2. This important finding prompted further investigations on the effect of LAF389 in endothelial cell proliferation and to compare it to fumagillin (**66**), a well-known anti-angiogenic natural product that inhibits MetAP2 enzyme [51,52]. The result of this study revealed that LAF389 displayed less pronounced endothelial specificity. Consequently, whereas fumagillin (**66**) and ovalicin (**67**) express selective inhibition of endothelial cell proliferation, the bengamides lack such selectivity, inhibiting the proliferation of all cell types tested, both endothelial and epithelial cells [48]. Nevertheless, additional biological studies with other bengamides revealed that bengamides M (**50**) and O (**52**) exhibited 10- to 20-fold selectivity toward MetAP1 which might imply a different antitumor activity profile (Table 3) [53,54].

**Table 3:** Inhibition of MetAP’s enzymatic activities by bengamides [IC_50_ (µM)].

Compound	MetAP1	MetAP2
Bengamide A (**38**)	1.9 ± 0.2	10.5 ± 3.8
Bengamide B (**39**)	29.3 ± 10.4	17.9 ± 7.9
Bengamide G (**44**)	26.8 ± 18.3	>50
Bengamide L (**49**)	37.1 ± 13.4	>50
Bengamide M (**50**)	5.4 ± 2.3	>50
Bengamide N (**51**)	40.2 ± 14.3	>50
Bengamide O (**52**)	2.7 ± 0.4	>50
Fumagillin (**66**)	NA	0.03
Ovalicin (**67**)	NA	0.0004

Having demonstrated that the methionine aminopeptidases were the direct biological targets for the bengamides, the next step in this research was to study their mode of action at the active site of these enzymes and to determine why this inhibition triggers an antiproliferative effect. To gain insight into the mode of action of the bengamides it is important to know the structure and function of the methionine aminopeptidases. These enzymes represent a unique class of metal dependent aminopeptidases that remove unblocked *N*-terminal initiator methionine on either peptides or proteins [55], both in a co-translational or post-translational mode [56], suggesting a role in regulating processes rather than general protein degradation [55]. The removal of the *N*-terminal methionine by MetAPs is a critical step in the maturation of many proteins and is essential for further amino terminal modifications [57]. Therefore, its inhibition has acquired a special importance since it has been demonstrated that MetAP2 is involved in the development of a certain number of tumors [58]. There are two isoforms of MetAP, types I and II, differing from one another by particular differential sequences [59,60]. Thus, the type II enzymes present an α-helical domain of 60 residues inserted in a surface loop of the *C*-terminal half of the molecule [61]. The proximity of this domain to the active site suggests that it is the key for the differentiation in specificity of the two classes. Also, the modifications due to the presence of *N*-terminal extensions further differentiate the enzymes [55]. Moreover, type I is further divided into types Ia, Ic (procaryotes) and Ic (only in eukaryotes). The eukaryotic MetAPs are differentiated from their prokaryotic counterparts by an additional *N*-terminal extension. The eukaryotic MetAP2 has two putative zinc finger motifs at the extreme *N*-terminus and a highly charged *N*-terminus with alternating polyacidic and polybasic stretches in a similarly sized segment. Although the catalytic domains of both MetAPs possess very similar structures, all the residues that form the methionine-binding pocket are different, but the shape of the pocket is conserved. Furthermore, MetAP2 also contains an inserted region contacting the catalytic domain in some of the same area covered by the connector of the type I [62]. These data support the proposal that MetAP types I and II may present a common functional role. From the biomedical point of view, MetAP2 has attracted much more attention than MetAP1 due to the discovery of MetAP2 and not MetAP1 as the biological target of some of the anti-angiogenic compounds, such as the aforementioned fumagillin (**66**) and ovalicin (**67**) [63], together with their synthetic analogues and other synthetic molecules [64]. Taking into account that methionine aminopeptidases have been identified as antitumor targets for these anti-angiogenic agents, it would be interesting to investigate the role of these enzymes in angiogenesis. However, despite numerous studies stressing the role of aminopeptidases in the formation of new blood vessels, it is still unclear what role MetAP2 plays in regulating angiogenesis [65].

**Figure 6:**
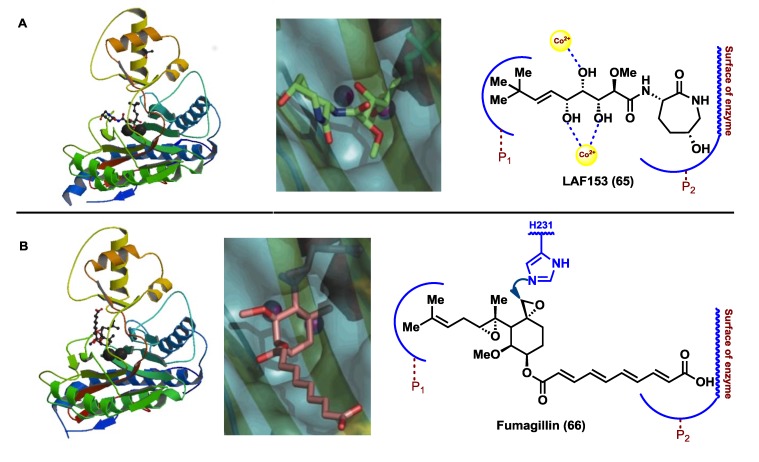
Crystal structures of the MetAP2-inhibitor complexes for LAF153 (PDB-YQYZ) and Fumagillin (PDB-1BOA) and mode of interactions at the active site. Note: this figure is adapted with permissions from [48]. Copyright © 2003, the American Society for Biochemistry and Molecular Biology.

The identification of MetAPs as the molecular targets for bengamides marked a turning point in the synthesis of potent and selective analogues that might assist not only to the generation of new anticancer leads but also to further investigate and unravel the role of MetAPs in cancer. As part of these investigations, Towbin *et al.* [48] were able to describe by X-ray studies the crystal structure of human MetAP2-bengamide complex, revealing that the real inhibitor was not LAF389 (**64**) but its non-acylated derivative, LAF153 (**65**) (Figure 5). This enzyme-substrate complex structure proved the mode of interaction of these bioactive compounds at the active site of the methionine aminopeptidases. The X-ray structure revealed a critical dinuclear metal center placed as a deep invagination in the surface of the enzyme [55]. On the other hand, the hydrophobic pocket P1, which contains the residues Phe-219, His-382 and Ala-414, in the innermost portion of the active-site, interacted with the terminal alkyl group of the olefin, while pocket P2, formed at the solvent-exposed surface by the residues of Leu-328, Phe-366 and His-231, hold the caprolactam ring. The coordination of the cobalt ions with the hydroxyl groups at C3, C4 and C5 occurred in a similar way to that observed for peptidic inhibitors of aminopeptidases [66], forming two octahedral geometric centers (Figure 6). Known MetAP2 inhibitors such as fumagilllin or ovalicin [52] exhibit a mode of inhibition that differs from that demonstrated for the bengamides, due to a covalent bond formed by nucleophilic attack of the amine group of a histidine residue (His-231) to one of the oxirane rings contained in these molecules (Figure 6). In contrast, the bengamides exert their inhibition as a result of multiple interactions. It should be noted that the mode of binding reported for LAF153 is similar to that of a bestatin-derived inhibitor of *Escherichia coli* MetAP [65].

**Figure 7:**
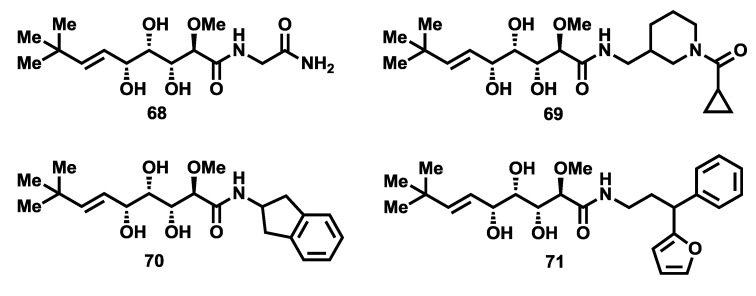
Molecular structures of bengamide analogues **68**–**71**.

More recently, in 2012, Ye *et al.* [67] uncovered further evidence regarding the role of the bengamides as inhibitors of MetAPs. In particular, they reported the X-ray structures of four bengamide analogues (**68**–**71**, Figure 7) in complex with *Hs*MetAP1 in the Mn (II) form, displaying a similar way of binding for these derivatives with respect to the natural bengamides except for the interaction at P2, due to the different amide structures. These four bengamide derivatives exhibited virtually similar IC_50_ values in their inhibition of the Mn (II) form of *Hs*MetAP1. Since they structurally differ at the amide moiety, this study suggests that the major linkage and stronger interactions at the active site derived from the *tert*-butylalkene and the triol fragments, although the modification of the caprolactam ring clearly affected the *in vitro* and *in vivo* activities, as reported earlier and will be described in Section 2.4.

With regard to the role and involvement of *Hs*MetAP1 in cancer, Liu *et al.* [68] elucidated the physiological function of this enzyme in cell proliferation by studying the X-ray structure of the complex *Hs*MetAP1/pyridine-2-carboxylic acid inhibitors [69,70], demonstrating that MetAP1 plays an important role in G2/M phase of the cell cycle. Further biological investigations in this field were accomplished to elucidate the key protein or proteins that were affected by the inhibition of methionine aminopeptidases and, as a consequence, produced the antitumor response. In this research, these authors identified and validated the proto-oncogene *c*-Src, involved in the development, growth, progression and metastasis of a number of human cancers [71], as a substrate for both MetAP1 and MetAP2 *in vivo* and *in vitro*. Thus, this research group showed that inhibition of MetAPs by the nonselective inhibitor bengamide A (**38**) altered the subcellular distribution of *c*-Src. This alteration significantly decreased its tyrosine kinase activity, and caused a remarkable delay in cell-cycle progression. Therefore, these results established a link between *c*-Src and MetAP and suggested that inhibition of MetAPs could indirectly impair the functions of *c*-Src and likely other oncogenes that are essential for tumor growth.

On the other hand, recent enzymatic studies has shown that depletion of MetAP2 by siRNA did not produce an inhibition of endothelial cell growth and, even more intriguing, these MetAP2-depleted endothelial cells remained responsive to inhibition by either bengamides or fumagillin [72]. In view of the foregoing, this data seems to indicate that MetAP2 is not required for endothelial cell proliferation and that MetAP2 may not be the target for the anti-angiogenic effect of the bengamides and fumagillin despite all the biological studies and evidence, exposed before, clearly supported the methionine aminopeptidases as the biological targets of the bengamides. The activity of these compounds in MetAP2-depleted endothelial cells is particularly puzzling because both classes of inhibitors have been co-crystallized with human MetAP2 [48,63]. According to the authors, one possible explanation to explain these controversial results is that another member of the MetAP family exists in humans that could be expressed in endothelial cells but able to be inhibited by both bengamide and fumagillin. Nonetheless, the majority of experimental evidence supports the notion that MetAP2 is essential for endothelial cell viability [73], although further biological studies are clearly required to gain insight into all these uncertainties.

Finally, the NF-κB (nuclear factor kappa B) inhibitory activity exhibited by the bengamides, recently discovered by Crews *et al.* [28], could be the responsible for their observed antitumor activities due to the close relationship between tumorigenesis and inflammation. In this biological study, qPCR analysis revealed that this inhibition affected the expression of the pro-inflammatory cytokines TNFα, IL6 and MCP-1. Thus, the effects of the bengamides on these key targets in the NF-κB pathway may contribute to their significant antitumor activity.

### 2.3. Chemical Synthesis of Natural Bengamides

**Scheme 2:**
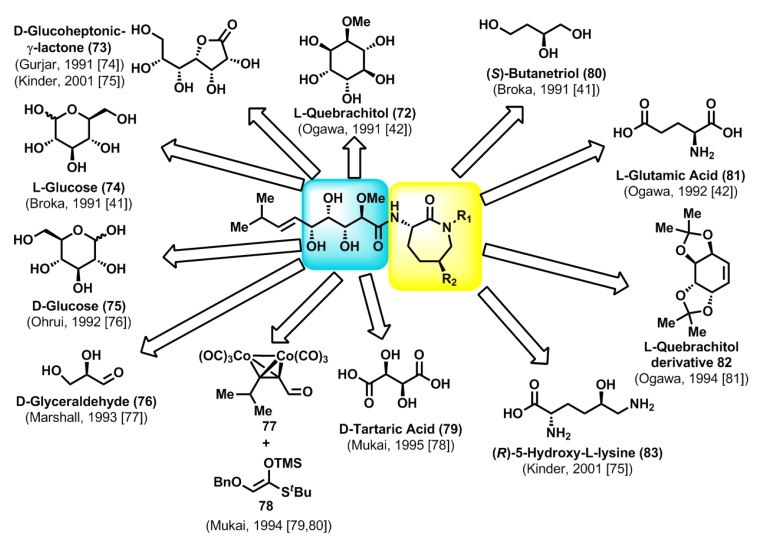
Total syntheses of bengamides in the period 1991–2001: general retrosynthetic analyses.

The novel molecular structures of the bengamides, coupled with their antitumor properties, rapidly propelled them to the forefront of chemical research. Thus, a few years after the discovery of the first bengamides by Crews and co-workers, Ogawa [42] reported the first total synthesis of bengamide E, which was accomplished starting from l-quebrachitol (**72**). The various total syntheses reported in the subsequent years after Ogawa’s first total synthesis employed monosaccharides such as d-gluconolactone (**73**) [74,75], l- and d-glucose (**74** and **75**) [41,76], d-glyceraldehyde (**76**) [77] or the d-tartaric acid (**79**) [78] as suitable chiral starting materials, taking into account the polyhydroxylated nature of the side chain. In contrast, the first synthesis conducted by Mukai was based on Mukaiyama-type aldol reactions starting from the achiral precursor **77** and the silylenol ether **78** [79,80], although subsequent improvements by the same author of his first synthesis of bengamide E were carried out by use of d-tartaric acid as starting material [78]. On the other hand, whereas the synthesis of bengamide E did not require the preparation of the caprolactam moiety, the synthesis of other members, such as bengamides A or B, required the stereoselective construction of this caprolactam fragment. In this sense, (*S*)-butanetriol (**80**) [41], l-glutamic acid (**81**) [42], l-quebrachitol derivative **82** [81] or (*R*)-5-hydroxy-l-lysine (**83**) [75] were the starting chiral materials used for the syntheses of the caprolactam moieties (Scheme 2). All the total syntheses reported in the literature during the period corresponding to 1991–2001 were reviewed by Kinder [82] and, therefore, are not described in detail in the present article.

**Scheme 3:**
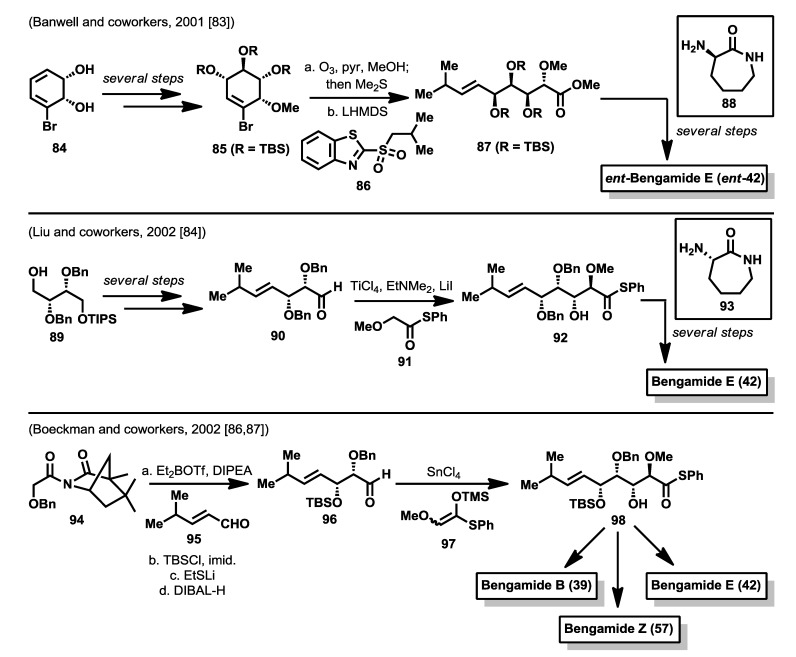
Total syntheses of bengamides by Banwell, Liu and Boeckman (2001–2002) [83,84,86,87].

Soon after the synthesis by Kinder *et al.* [75] reported in 2001, Banwell and McRae reported a different synthesis of the enantiomer of bengamide E [83] via a chemoenzymatic approach, in which starting from 1,2-dihydrocatechol **84**, readily available by microbial oxidation of bromobenzene, compound **85** was prepared, after several steps involving oxirane ring formation, oxirane ring opening and protecting groups manipulations. Ozonolysis of this cyclohexene derivative **85**, conducted in CH_2_Cl_2_-MeOH, afforded the corresponding aldehydic ester, which was exposed to the anion of sulfone **86** to yield *E* alkene **87** almost exclusively. Ester **87** was then hydrolyzed and the resulting acid coupled with cyclo-l-Lysine **88** to afford, after silyl ether cleavage, the enantiomer of bengamide E (*ent*-**42**). One year later, Liu and coworkers published the synthesis of bengamide E starting from alcohol **89**, obtained from diisopropyl d-tartrate and based on an aldol reaction strategy for the construction of the polyol system [84]. Thus, after several steps in which the double bond was introduced via a Julia-Kocienski reaction, the aldehyde **90** resulting from **89** was subjected to an aldol reaction with thioester **91**, under Annuziata conditions [85], to obtain a 1:1 mixture of the *anti* diastereoisomers. After separation of both isomers, the correct *anti* diastereoisomer (compound **92**) was coupled with cyclo-l-lysine (**93**), followed by Birch reduction of the benzyl ether protecting groups to obtain bengamide E (**42**). As in the Mukai’s synthesis, the aldol approach to the bengamides is marked by the absence of stereoselectivity (Scheme 3).

A new approach to the bengamides, based again on aldol reactions, was conducted by the Boeckman’s group who described the total synthesis of bengamides B (**39**), E (**42**) and Z (**57**) [86,87]. For these synthesis, the C10 side chain was entirely constructed through sequential *syn* and *anti* asymmetric aldol reactions from the α,β-unsaturared aldehyde **95**. The first aldol reaction was carried out with the chiral acetimide **94**, which, after treatment with Et_2_BOTf and DIPEA, was reacted with **95** to furnish the corresponding *syn* aldol product in excellent diastereoselectivity (>24:1). The elaboration of this product for the second aldol reaction led to the aldehyde **96** which was subjected to the action of the silylenol ether **97** in the presence of SnCl_4_ to obtain compound **98** in 73% yield and 11.5:1 diastereomeric ratio. This compound **98** represents the common intermediate for the syntheses of bengamides B, E and Z by direct coupling of the thioester **98** with the corresponding 2-aminocaprolactams. For the syntheses of the caprolactams contained in bengamides B and Z, the authors employed D-aspartic acid as the chiral starting material that allowed the construction of (*R*)-2-bromobutane-1,4-diol as a key intermediate for their syntheses. However, for the bengamide B synthesis, the final benzyl ether deprotection, by the action of Na in NH_3_, produced the cleavage of the fatty acid ester present in the caprolactam fragment, resulting in a need for a strategy modification to build the polyketide side chain. This modification consisted of the replacement of the benzyl ether group at C4 for a 2-naphtylmethyl group, which was cleaved at the end of the synthesis by sequential treatment with DDQ and PPTS that did not affect the integrity of the fatty acid ester moiety, allowing the completion of the synthesis (Scheme 3).

**Scheme 4:**
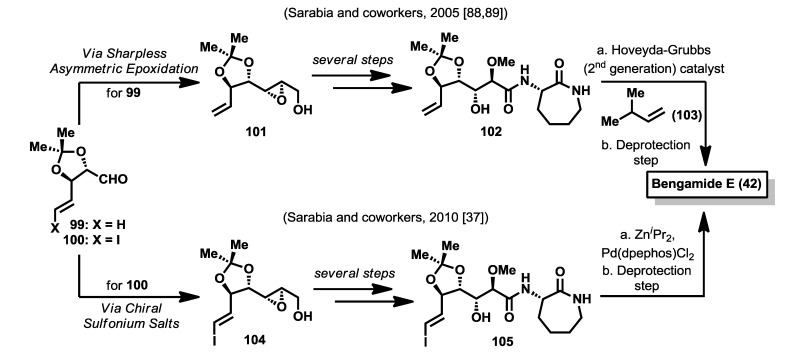
Total syntheses of bengamide E by Sarabia (2005 and 2010) [37,88,89].

In 2005, Sarabia *et al.* [88,89] reported a first synthesis of bengamide E based on three key steps that were: (1) a cross olefin metathesis to introduce the terminal alkyl group; (2) an epoxide ring opening reaction to construct the polyketide side chain; and (3) an amide bond formation to introduce the caprolactam moiety. Thus, starting from aldehyde **99** and, through the Sharpless methodology of asymmetric epoxidation, epoxy alcohol **101** was prepared in a complete stereoselective fashion. After a regioselective ring opening reaction of **101** with MeOH, chemoselective oxidation of the primary alcohol to the acid and coupling with **93** provided **102** which was subjected to a cross olefin metathesis with olefin **103**, mediated by the 2nd generation Hoveyda-Grubbs catalyst, to afford the *E*-substituted olefin in good yield and complete stereoselectivity. Bengamide E was finally synthesized after a deprotection step of the acetal group (Scheme 4). In a second and improved synthesis by the same authors in 2010 [37], and as an application of the group’s methodology in the field of amide-stabilized sulfur ylides [90], aldehyde **100** was reacted with a chiral sulfonium salt to obtain in good yield and excellent diastereoselectivity (>98%) an epoxy amide that was reduced to epoxy alcohol **104**. From this epoxy alcohol, the methodology proceeded in a similar manner as the previous synthesis, although the processes of ring opening reaction and the formation of the substituted olefin were remarkably improved with respect to the first synthesis. Thus, the ring opening reaction of **104** was carried out by treatment of MeOH in the presence of B(OMe)_3_, as an extension of the methodology developed of Miyashita [91]. After oxidation and coupling reactions of the ring opening product, the resulting vinyl iodide **105** was reacted with diisopropylzinc, catalyzed by palladium (0), to install the terminal olefinic substituent and, after a deprotection step, bengamide E was prepared in a shorter and more efficient route compared with the first one described in 2005 (See Table 4). As will be discussed in the following section, one of the advantages of this latter synthetic strategy, as others described in the literature, is the versatility and convergency that allows one to generate bengamide analogues modified at various parts of the structure by suitable use of different reagents employed along the synthetic pathway previously delineated.

**Table 4:** Synthesis of natural bengamides: A summary.

Bengamide	Author/Year [Ref.]	Starting Material	Number of Steps ^1^	Overall Yield ^2^ (%)
E (**42**)	Ogawa/1991 [42]	l-quebrachitol	15	0.40%
E (**42**)	Broka/1991 [41]	l-glucose	16	11.20%
B (**39**)	Broka/1991 [41]	l-glucose	16	5.90%
E (**42**)	Ohrui/1992 [76]	d-glucose	17	0.80%
A (**38**)	Ogawa/1992 [40]	l-quebrachitol	16	0.24%
E (**42**)	Marshall/1993 [77]	d-mannose	12	10.71%
E (**42**)	Mukai/1994 [79]	Isobutyraldehyde	18	3.06%
B (**39**)	Ogawa/1994 [81]	l-quebrachitol	16	0.09%
E (**42**)	Mukai/1995 [80]	Isobutyraldehyde	18	4.70%
E (**42**)	Mukai/1995 [78]	DIDT ^3^	16	5.00%
B (**39**)	Kinder/2001 [75]	d-gluconolactone	7	7.21%
E (**42**)	Kinder/2001 [75]	d-gluconolactone	7	5.43%
*ent*-E (*ent***42**)	Banwell/2001 [83]	Bromobenzene	12	5.70%
E (**42**)	Liu/2002 [84]	DIDT ^3^	10	9.74%
B (**39**)	Boeckman/2002 [86,87]	Ethyl 2-hydroxyacetate	12	10.86%
E (**42**)	Boeckman/2002 [86]	Isobutyraldehyde	10	23.56%
Z (**57**)	Boeckman/2002 [86]	Isobutyraldehyde	10	17.86%
E (**42**)	Sarabia/2005 [89]	Diethyl d-tartrate	18	2.85%
E (**42**)	Sarabia/2010 [37]	Diethyl d-tartrate	14	4.15%
E (**42**)	Li/2013 [92]	d-glucose	16	4.00%
E (**42**)	Prasad/2013 [93]	Diethyl d-tartrate	12	7.05%

^1^ The number of steps is for the longest linear sequence; ^2^ the overall yield is for the longest linear sequence, not considering the synthesis of the caprolactam fragment for the cases of bengamides A, B and Z; ^3^ DIDT = Diisopropyl d-tartrate.

**Scheme 5:**
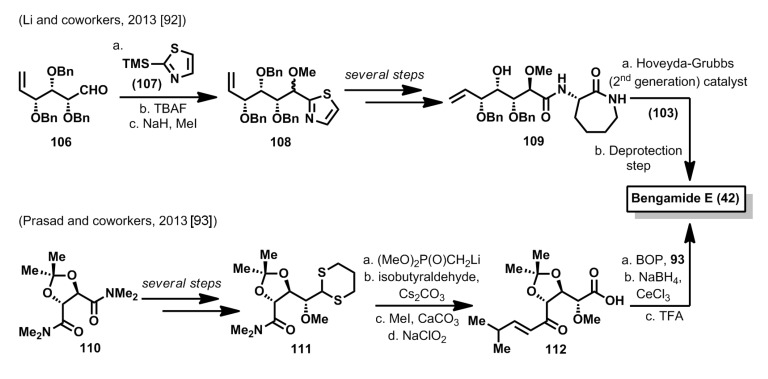
Total syntheses of bengamide E by Li and Prasad (2013) [92,93].

Two more total syntheses of bengamide E have been reported in the last year. In the first one, by Li *et al.* [92], bengamide E, together with some new analogues, were synthesized from hex-5-enal derivative **106**, which was prepared from α-methyl-d-glucopyranoside through a known synthetic sequence. Thus, by use of the Dondoni methodology [94], aldehyde **106** was reacted with thiazol derivative **107** to afford a 1:1 diastereomeric mixture of compound **108**, after TBAF treatment and methylation of the resulting alcohol. After subsequent transformation of the thiazol moiety into the acid and coupling with lactam **93**, an olefin cross metathesis provided the bengamide E precursor, which was converted to the final product after deprotection steps (Scheme 5). A second synthesis of bengamide E has been reported by Prasad *et al.* [93] starting from the bis(dimethylamide) of d-tartaric acid **110**, whose desymmetrization by reaction with the anion of 1,3-dithiane, led to compound **111**, after a reduction and a methylation steps [95]. After the synthesis of a β-ketophosphonate, a Horner-Wadsworth-Emmons olefination allowed the stereoselective introduction of the substituted olefinic fragment. Finally, dithiane oxidation that led to **112**, followed by a caprolactam coupling, reduction of the ketone with NaBH_4_/CeCl_3_ and final acidic deprotection allowed the completion of the synthesis of bengamide E (Scheme 5).

Throughout this section the total syntheses of natural bengamides has been reviewed. As a summary, Table 4 comprises all the synthesis achieved up to now, indicating the number of steps and overall yields for comparative purposes. In this sense, it highlights the fact that over the years more efficient and shorter syntheses of the bengamides have been reported and has allowed preparation not only of natural bengamides in sufficient amounts for biological and clinical assays, but also the synthesis of analogues for identification of more potent bengamides from the biological point of view, as will be discussed further in the following section.

### 2.4. Synthesis of Bengamide Analogues and Biological Evaluation

**Figure 8:**
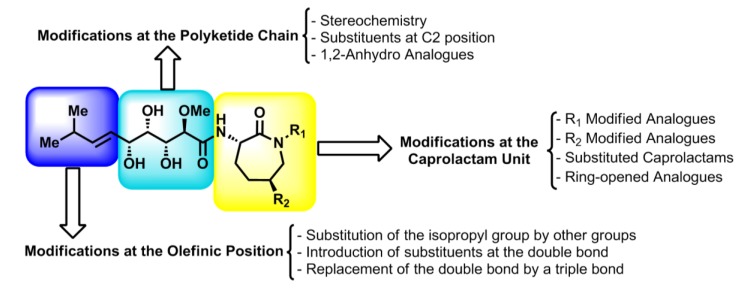
Designed bengamides: structural fragments amenable to modifications.

The structures of the bengamides are amenable to several modifications that could lead to the establishment of structure-activity relationships (SAR), which are essential for the design and synthesis of new potential drug candidates. Thus, configurational modifications of the different stereocenters located at the polyketide chain, changes of the substituent located at the terminal olefinic position or introduction of modified caprolactams have been reported by different authors (Figure 8), contributing to the formulation of more potent bengamide derivatives that are detailed throughout this section.

**Scheme 6:**
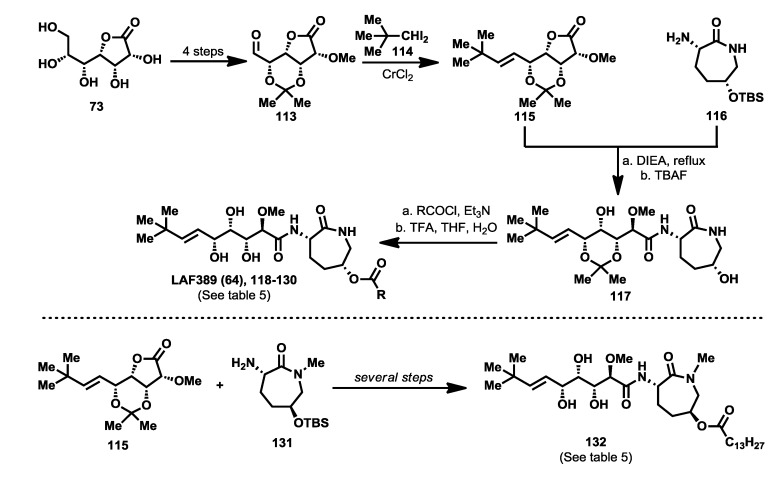
Syntheses of bengamide analogues by Kinder (2001) [46].

The first bengamide analogues were synthesized by Kinder’s group in 2001 [46], consisting of ester-modified derivatives of bengamide B, that led to new bengamides with impressive *in vitro* and *in vivo* antitumor activities but with poor water solubility. In order to address this drawback, several key modifications were considered such as simplification of the lactam moiety and modification of the alkyl group at the olefinic position. The synthetic pathway used for the preparation of these analogues was based on the previous syntheses of bengamides B and E reported by the same author, starting from d-gluconolactone **73** and replacing the terminal isopropyl group by a *tert*-butyl [75]. Thus, from aldehyde **113**, readily prepared from **73**, the introduction of the substituted *tert*-butyl *E*-olefin was achieved by means of a Takai-Utimoto olefination with 1,1-diiodoneopentane (**114**) [96] to obtain key lactone **115** in a reasonable good yield (63%). The reaction of this lactone with caprolactam **116**, prepared from the commercially available (5*R*)-5-hydroxy-l-lysine, afforded bengamide derivative **117**, which by reaction with either an acid chloride or a carboxylic acid, followed by acetal cleavage, provided an array of bengamide A analogues (**118**–**130**), including the aforementioned LAF389 (**64**). Taking into account that all these analogues possess the opposite configuration at the C-5′ position of the caprolactam ring with respect to the natural bengamides, these authors prepared similarly the *tert*-butyl analogue of bengamide B (**132**) by reaction of lactone **115** with the amino caprolactam **131**, which was prepared from **116** (Scheme 6).

The cytotoxicity of these bengamide analogues was evaluated by measurement of their antiproliferative activities against MDA-MB-435 human breast carcinoma cells (*in vitro* and *in vivo*) (Table 5), and as a result, some important conclusions related to the structure/activity relationship were established. Analogue **64**, which replaces the myristate ester by a cyclohexane carboxylic ester and whose configuration at C5′ was inverted respected to bengamide B, was the most potent analogue of the series (both *in vitro* and *in vivo*). This result delivered interesting outcomes concerning both the configuration of the lactam at the 5′-position together with the increased water solubility due to the substitution of the myristate chain with fewer lipophilic groups. Additionally, compounds **64** and **124** were found to cause tumor regression with minor losses in body weight, with compound **64**, corresponding to LAF389, being the most potent *in vivo*, producing 29% tumor regression at 100 µmol/kg. Interestingly, the bengamide B analogue **132** displayed similar *in vitro* and *in vivo* potency as its natural counterpart, bengamide B [46].

**Table 5:** Cytotoxic activities (IC_50_, μM) of ester modified analogues of bengamides.

Bengamide	R-	MDA-MB-435	Bengamide	R-	MDA-MB-435
**118**	CH_3_(CH_2_)_12_-	<0.01	**124**	PhCH_2_CH_2_-	0.01 ± 0.01
**119**	Cyclopentyl	0.13 ± 0.01	**125**	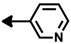	5.85 ± 0.01
LAF389 (**64**)	Cyclohexyl	0.04 ± 0.00	**126**	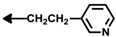	0.46 ± 0.04
**120**	Cycloheptyl	0.02 ± 0.01	**127**	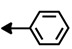	0.37 ± 0.01
**121**	*c*-HexylCH_2_-	0.06 ± 0.00	**128**	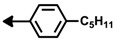	0.02 ± 0.01
**122**	PhCH_2_-	0.26 ± 0.04	**129**	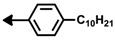	0.03 ± 0.04
**123**	*E*-PhCH=CH-	0.02 ± 0.00	**130**	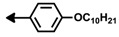	0.01 ± 0.00
**132**	-	0.01 ± 0.00	B (**39**)	-	0.012 ± 0.003

In light of these promising results, in particular for LAF389 (**64**), Novartis developed in 2003 a large-scale and optimized synthesis of this analogue, which allowed its preparation on more than a 100 g scale for preclinical and clinical studies [49]. Thus, LAF389, as a new potential antitumor lead, was launched to clinical trials. However, the lack of clinical activity and the finding of unpredictable cardiovascular toxicity caused the compound to be halted for further clinical investigation and future clinical assays were canceled [97]. A possible reason for its failure may be due to its lack of selectivity in the inhibition of both MetAP1 and MetAP2, as well as its poor bioavailability, all this despite the potent cytotoxicity and significant *in vivo* activity exhibited by LAF389. Nevertheless, these results prompted the search for new, more potent analogues with better selectivity and solubility properties.

**Scheme 7:**
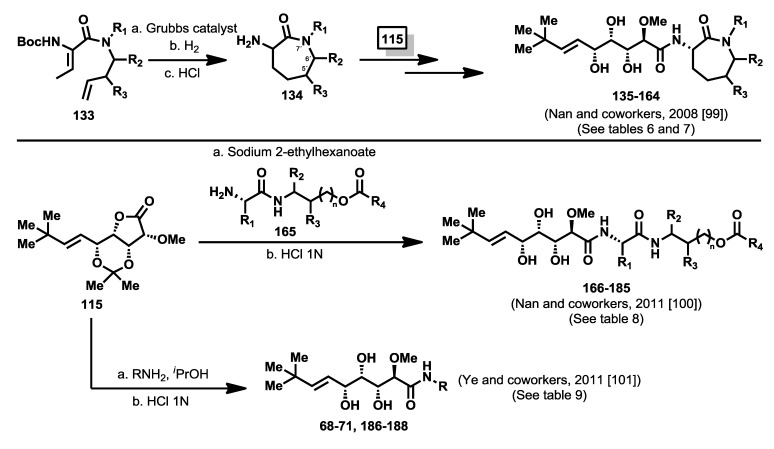
Syntheses of caprolactam-modified bengamide analogues [99,100,101].

In 2008, Nan and co-workers, following the ring-closing metathesis methodology described previously by them for the synthesis of functionalized amino caprolactams [98], described the synthesis of new bengamide analogues modified at the caprolactam moiety at positions 5′-, 6′-, and 7′- by reaction of lactone **115** with amino caprolactams type **134**, obtained from the diene precursors **133**, with an overall yield between 25% and 70% [99] (Scheme 7). The bengamide library (**135**–**164**) was evaluated against MDA-MB-435 human breast carcinoma cells (Table 6 and Table 7). The biological results showed that whereas modifications at position 5′ apparently did not seem to affect to the activity (which is in accordance with Novartis studies of modifications in this position), the 6′-substituted derivatives were well tolerated. Interestingly, *N*-substituted bengamide analogues exhibited an increase in activity when the chain length of the R group was enlarged, with the best results provided for the two-carbon chain length (case of analogue **142** with a IC_50_ of 17 nM). However, these effects were not clear when considering aryl containing analogues (cases of **144**–**146**), and in fact it decreased for simple *N*-alkyl amines. This study resulted in the identification of a new potent analogue, the *N*-acetyl derivative **142** [99], which displayed greater activity (17 nM) and greater water solubility (10 mg/mL) than LAF389 (1.0 mg/mL) (Table 6). As the data reflect in Table 7, substitution at C5′ and C6′ positions led to less potent analogues than those showed in Table 6 with the exception of the diastereomeric mixture of LAF385 (**64**) and its C5′ epimer (**64′**) whose cytotoxicity was similar to pure **64**. A more profound modification of the caprolactam moiety led Nan and coworkers [100] to the discovery in 2011 of a series of novel ring-opened bengamide analogues (**166**–**185**) in which the caprolactam ring was replaced by a linear peptidic chain (Scheme 7). The biological evaluation of these new 20 analogues against MDA-MB-435 human breast carcinoma cells revealed compound **172**, as one of the most potent antitumor compounds belonging to the bengamide family, with an IC_50_ value of 4.0 nM and improved water solubility (1.0 mg/mL), when compared with the solubility of bengamide B (0.002 mg/mL) (Table 8). This work has delivered relevant results that could be very useful for further SAR studies and novel modified analogues. In the same year, Ye *et al.* [101] outlined the design of a new type of bengamide derivatives displaying a strong inhibition of *Mycobacterium tuberculosis* methionine aminpeptidases, but weak inhibition against human methionine aminopeptidases. While the triol moiety and the alkene substituent were kept to maintain the interaction with the two metal ions at P1, the caprolactam moiety was replaced with various amide moieties to study their interactions in P2 pocket. The syntheses of these analogues were performed by coupling of lactone **115** with several amines to obtain, after a deprotection step, the analogues **68**–**71** and **186**–**188** (Scheme 7). Their inhibitory activities were tested toward *Mt*MetAP1a and *Mt*MetAP1c activated by the metal ions Co^2+^, Mn^2+^, Ni^2+^ and Fe^2+^. All compounds were almost inactive against the Co^2+^ or Fe^2+^ forms of *Mt*MetAP1c, and completely inactive against the Ni^2+^ one, proving the selectivity for the Mn^2+^ form in the low μM range. Additionally, compound **188** exhibited the best antitubercular activity against both replicating and non replicating *Mycobacterium tuberculosis*, although their activities against human MetAPs were not completely suppressed according to the evaluation against human K562 cells that showed significant activity for some of these bengamide analogues (Table 9).

**Table 6:** Cytotoxic activities (IC_50_, μM) of caprolactam-modified analogues of bengamides by Nan *et al.* (2008) [99].

Bengamide	R_1_ (R_2_, R_3_=H)	MDA-MB-435	Bengamide	R_1_ (R_2_, R_3_=H)	MDA-MB-435
**135**	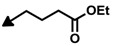	1.120 ± 0.240	**143**	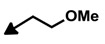	0.396 ± 0.046
**136**	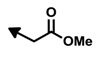	0.25 ± 0.021	**144**	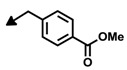	0.236 ± 0.064
**137**	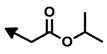	0.424 ± 0.017	**145**	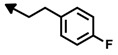	0.557 ± 0.129
**138**	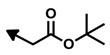	0.275 ± 0.005	**146**	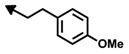	0.269 ± 0.041
**139**	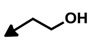	0.202 ± 0.005	**147**	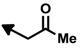	0.276 ± 0.029
**140**	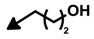	0.424 ± 0.068	**148**	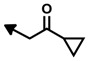	1.286 ± 0.226
**141**	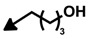	0.358 ± 0.071	**149**	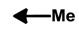	0.626 ± 0.234
**142**	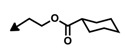	0.017 ± 0.008	**150**	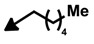	0.781 ± 0.160

**Table 7:** Cytotoxic activities (IC_50_, μM) of caprolactam-modified analogues of bengamides by Nan *et al.* (2008) [99].

Bengamide	R_1_	R_2_	R_3_	MDA-MB-435
**64** + **64′**	H	H	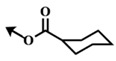	0.040 ± 0.002
**151**	H	H	AcO	2.092 ± 0.231
**152** (cis)	H	H	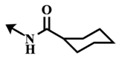	2.393 ± 0.382
**153** (trans)	H	H	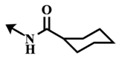	2.013 ± 0.180
**154**	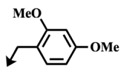	H	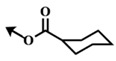	0.712 ± 0.016
**155**	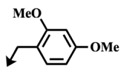	H	AcO	0.364 ± 0.059
**156** (more polar)	OH	H	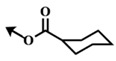	0.078 ± 0.010
**157** (less polar)	OH	H	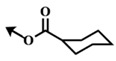	0.312 ± 0.014
**158**	H	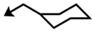	AcO	0.466 ± 0.033
**159**	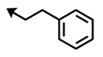	H	H	0.287 ± 0.039
**160**	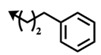	H	H	1.306 ± 0.476
**161**	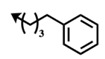	H	H	0.275 ± 0.028
**162** + **162′** (2′*S* + 2′*R*)	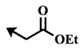	H	H	0.305 ± 0.017
**162** (2′*S*)	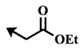	H	H	0.141 ± 0.009
**163** (2′*R*)	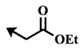	H	H	1.617 ± 0.274
**164**	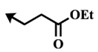	H	H	2.113 ± 0.532

**Table 8:** Cytotoxic activities (IC_50_, nM) of ring-opened analogues of bengamides by Nan *et al.* (2011) [100].

Bengamide	R_1_	R_2_	R_3_	R_4_	N	MDA-MB-435	Bengamide	R_1_	R_2_	R_3_	R_4_	n	MDA-MB-435
**166**	Me	H	H	CH_3_(CH_2_)_12_	0	19 ± 4	**176**	Me	H	H	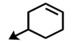	0	16 ± 37
**167**	Me	H	H	CH_3_(CH_2_)_4_	0	10 ± 1	**177**	Me	H	H	PhCH_2_CH_2_-	0	12 ± 2
**168**	Me	H	H	*i*-Pr	0	293 ± 80	**178**	Me	H	H	Cyclopentyl	0	9 ± 7
**169**	Me	H	H	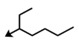	0	125 ± 35	**179**	Me	H	H	Cyclopropyl	0	675 ± 15
**170**	Me	H	H	*tert*-Bu	0	154 ± 37	**180**	Me	H	H	Cyclobutyl	0	20 ± 13
**171**	Me	H	H	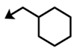	0	31 ± 14	**181**	Me	H	H	Cyclohexyl	0	31 ± 14
**172**	Me	H	H	Cyclohexyl	1	4 ± 11	**182**	*i*-Bu	H	H	Cyclohexyl	0	125 ± 9
**173**	Me	CO_2_Et	H	Cyclohexyl	0	65 ± 6	**183**	*n*-Bu	H	H	Cyclohexyl	0	157 ± 8
**174**	Me	H	Me	Cyclohexyl	0	17 ± 11	**184**	*i*-Pr	H	H	Cyclohexyl	0	105 ± 10
**175**	*sec*-Bu	H	H	Cyclohexyl	0	208 ± 76	**185**	BocNH(CH_2_)_4_	H	H	Cyclohexyl	0	141 ± 22

**Table 9:** Activities (IC_50_, μM) against *Mt*MetAPs and k56 cells of amide-modified analogues of bengamides by Ye *et al.* (2011) [101].

Bengamide	R	*Mt*MetAP1a				*Mt*MetAP1c				K562
		Co	Mn	Ni	Fe	Co	Mn	Ni	Fe	
**186**	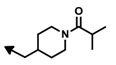	45	68	141	67	>250	1.3	>250	179	>333
**69**	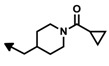	88	187	>250	110	>250	0.96	>250	>250	>333
**71**	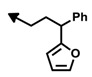	6.0	11	26	5.5	39	0.40	150	61	79.6
**187**	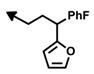	8.1	12	80	6.7	52	0.20	>250	68	96.5
**70**	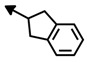	31	11	140	18	>250	0.76	>250	201	>333
**68**	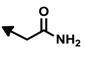	21	49	114	14	50	0.62	120	3.7	>333
**188**	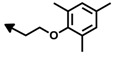	7.9	6.9	33	4.5	75	0.54	>250	42	37.8

**Figure 9:**
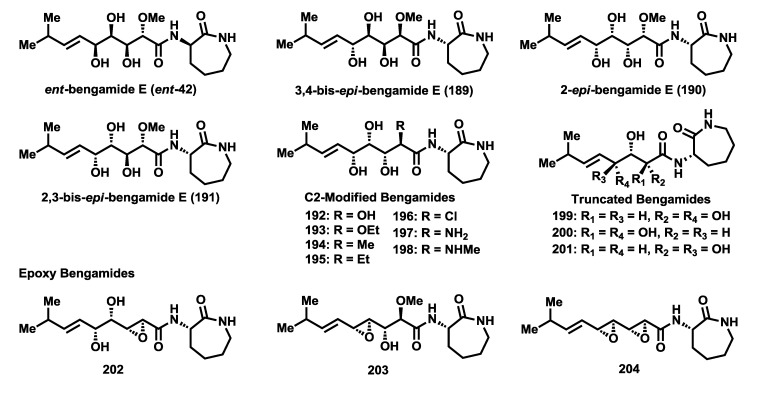
Polyketide-modified bengamides.

The essential role that the polyhydroxylated chain of the bengamides plays in their interaction with methionine aminopeptidases was demonstrated with the synthesis of various polyketide-modified analogues and their corresponding biological evaluations. The effect of the configuration of the polyketide chain on the cytotoxic potency was evaluated through the 3,4-*epi*-, 2-*epi*- and 2,3-*epi*-bengamide E analogues (**189**–**191**) [92,102,103] (Figure 9). Together to all these stereo-isomers analogues of bengamide E, the enantiomer of the natural product (***ent*-42**) was prepared and biologically tested [83]. It was also possible to assess the biological significance of the methoxyl group at C2 position through the synthesis of the C2-modified analogues **192**–**198** [89,103]. Finally, the influence of the constitution of the polyketide chain on the biological activity was similarly studied through truncated analogues **199**–**201** [92] and the epoxy derivatives **202**–**204** [103], which were designed and prepared as potential fumagillin-bengamide hybrids. The biological results obtained by cytotoxic evaluations of all these analogues against a panel of different tumor cell lines revealed that this polyketide chain was essential to retain the biological activity of this class of compounds and that any change, configurational or constitutional, of this chain produced the complete loss of cytototoxic potency. Thus, Table 10 displays only the most active analogues of this series. Consequently, it was clearly demonstrated that the polyol system was not amenable to modification, supporting the notion of the strong involvement of this polyketide chain in binding with the active site of the MetAP enzymes through metal ion coordination with the hydroxyl groups.

**Table 10:** Cytotoxic activities (IC_50_, μM) against different tumor cells lines of selected polyketide-modified bengamides.

Bengamide	Cell Line	IC_50_	Bengamide	Cell Line	IC_50_
**190**	HT29	85.2 ± 17.0	**197**	HT29	38.5 ± 4.8
	MDA-MB-435	100.2 ± 13.3		MDA-MB-435	70.7 ± 12.0
	HT1080	93.2 ± 8.1		HT1080	30.2 ± 6.1
	HL60	60.3 ± 15.1		HL60	25.3 ± 5.2
	BAEC	69.7 ± 3.2		BAEC	28.9 ± 7.2
**192**	HCT-116	25.49	**198**	HT29	10.6 ± 1.2
	MDA-MB-435	11.36		MDA-MB-435	12.5 ± 3.0
	MCF-7	8.35		HT1080	5.8 ± 0.1
	-	-		HL60	9.2 ± 50.6
	-	-		BAEC	4.1 ± 1.3

**Scheme 8:**
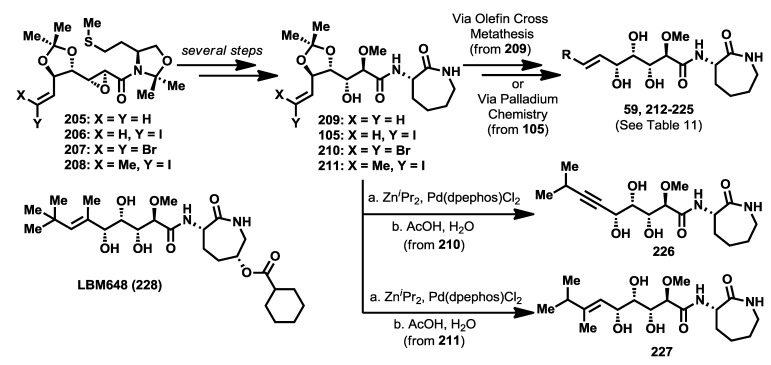
Synthesis of olefinic-modified bengamide analogues.

A final set of analogues, modified at the terminal olefinic position, was synthesized and biologically evaluated by Sarabia and co-workers [104]. Apart from the bengamide analogue LAF389 (**64**) and other related caprolactam-modified compounds, in which the isopropyl moiety was replaced with a *tert*-butyl group, no other structural modifications were undertaken in this region of the molecule. Considering the binding mode of bengamides at the MetAP active site in which a hydrophobic interaction occurs at the P1 pocket, structural modifications in this region could reinforce this hydrophobic interaction and, therefore, have a positive effect upon the antitumor activity. To this aim, an array of bengamide analogues modified at this position was synthesized by use of an earlier described methodology based on chiral sulfur ylides [90]. Thus, chiral epoxy amides **205**–**208**, efficiently prepared by this methodology, carried proper functionalization at the olefinic position for a subsequent introduction of different alkyl groups. These epoxy amides were transformed into the products **105** and **209**–**211**, which, through olefin cross metathesis or palladium-mediated couplings (Suzuki [105], Sonogashira [106] and Negishi [107]), led to the preparation of bengamide analogues **212**–**227**. In addition to this array of bengamide E analogues, the olefin-modified analogue LBM648 (**228**) was also prepared by a different research group [72,108] (Scheme 8).

In order to evaluate the cytotoxic properties of this new library of bengamide E analogues, IC_50_ values were determined first against HT29 human colon adenocarcinoma cell line and later against other additional cancer cell lines, namely MDA-MB-231 (human breast carcinoma), HT1080 (human fibrosarcoma) and HL60 (human promyyelocytic leukemia), as well as against a primary culture of non-transformed bovine aorta endothelial (BAE) cells (Table 11), establishing a more complete and detailed antiproliferative profile for the most active members with fumagilin as a positive control. The data summarized in Table 11 suggested that an increasing of the polyketide chain at the olefinic position resulted in a complete loss of cytotoxic activity. More exciting results were obtained for analogues bearing a cyclic group, emphasizing cyclopentyl analogue **219**, which exhibited the best cytotoxic result among all the evaluated analogues, with a fourfold improvement in antitumor activity over bengamide E. It is interesting to highlight how essential the substitution at the olefinic position is for antitumor activity, as demonstrated by the methylene analogue **212**, which was completely devoid of activity, as well as the importance of the molecular size at this position, proved with analogues **214**–**216** and **221** which were similarly inactive.

**Figure 10:**
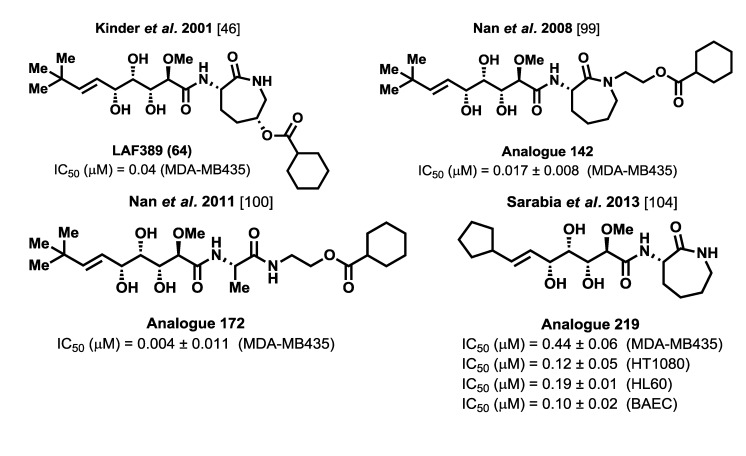
Representative bengamide analogues with potent antitumor activities [46,99,100,104].

In total, 111 analogues of the bengamides have been synthesized thus far and their biological activities evaluated. Among these analogues, the most potent and promising analogues are depicted in Figure 10. Compilation of all the biological information obtained from the prepared analogues provides an extensive structure-activity relationship study that allows the establishment of a well-defined pharmacophore map for the bengamides and provides opportunities for the development of new generations of more active and promising analogues.

**Table 11:** Cytotoxic activities (IC_50_, μM) against different tumor cells lines of bengamide E analogues modified at the terminal olefinic position.

Bengamide	R-	Cell Line	IC_50_	Bengamide	R-	Cell Line	IC_50_
**212**	H-	HT29	>100	**220**	Cyclohexyl	HT29	62.5
**213**	Ph-	HT29	68.8	**221**	*E*-ChxCH=CH-	HT29	100
**214**	(CH_3_)_2_CHCH_2_-	HT29	38.5	**222**	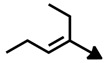	HT29	33.7 ± 3.0
**215**	(CH_3_)_3_CCH_2_-	HT29	100	**223**	TMSC≡C-	HT29	33.3 ± 10.0
**216**	*E*-(CH_3_)_3_CH=CH-	HT29	61	**224**	Cyclopropyl-	HT29	2.1 ± 0.4
						MDA-MB-435	3.7 ± 0.5
						HT1080	1.1 ± 0.5
						HL60	2.1 ± 0.4
						BAEC	1.6 ± 0.5
**217**	(CH_3_)_3_-	HT29	0.087 ± 0.2	**225**	CH_3_C(=CH_2_)-	HT29	16.9 ± 3.7
		MDA-MB-435	2.2 ± 0.1			MDA-MB-435	15.2 ± 2.5
		HT1080	0.25 ± 0.05			HT1080	1.7 ± 0.7
		HL60	1.1 ± 0.2			HL60	6.3 ± 0.2
		BAEC	0.17 ± 0.01			BAEC	2.3 ± 0.2
**218**	I-	HT29	14.8 ± 2.0	**226**	See Scheme 8	HT29	2.2 ± 0.4
		MDA-MB-435	14.2 ± 1.4			MDA-MB-435	5.7 ± 2.0
		HT1080	3.6 ± 1.0			HT1080	0.5 ± 0.1
		HL60	7.1 ± 0.5			HL60	1.7 ± 0.1
		BAEC	3.9 ± 0.4			BAEC	1.5 ± 0.2
**219**	Cyclopentyl-	HT29	0.22 ± 0.05	**227**	See Scheme 8	HT29	6.6 ± 0.7
		MDA-MB-435	0.44 ± 0.06			MDA-MB-435	11.1 ± 3.0
		HT1080	0.12 ± 0.05			HT1080	1.21 ± 0.13
		HL60	0.19 ± 0.0			HL60	5.5 ± 1.0
		BAEC	0.10 ± 0.02			BAEC	2.4 ± 1.3
**228**	See Scheme 8	A549	0.74 ± 0.2	-	-	-	
		HI299	0.99 ± 0.76				
		HCT116	0.14 ± 0.15				
		HUVEC	0.14 ± 0.15				

## 3. Chemistry and Biology of the Bengazoles

### 3.1. Discovery and Structural Elucidation of Bengazoles

**Figure 11:**
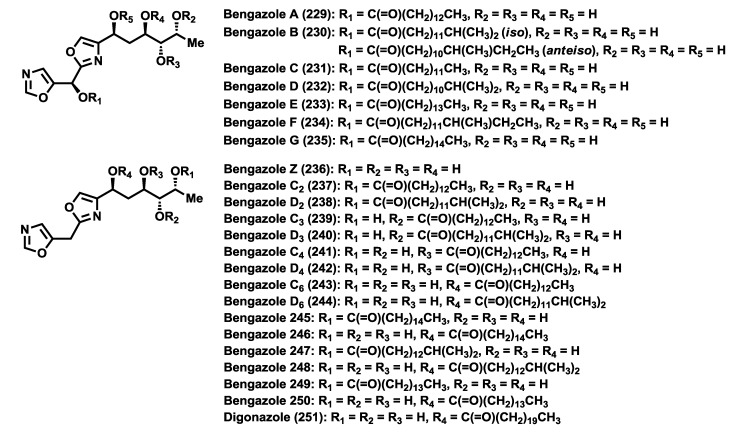
Molecular structures of the bengazoles.

The second most common secondary metabolites found in *Jaspis* species are the bengazoles (Figure 11), a group containing both bis(oxazolyl)methanol compounds (such as bengazole A–G), and bis(oxazolyl)-methane compounds (such as bengazoles Z, C_2_–D_6_ and digonazole), substituted by either a tetrahydroxylated or alkoxylated six-carbon chain [2]. The history of the bengazoles commences in 1988 when Crews and co-workers [34] isolated, from a huge amount of *Jaspidae* sponge collected from new locations in the Benga Lagoon, Fiji, two unusual oxazoles, bengazoles A (**229**) and B (**230**). Subsequently, in 1993, Crews *et al.* [109] discovered, in a Papua New Guinea specimen an inseparable mixture of seven new bengazoles (C_2_, D_2_, C_3_, D_3_, C_4_, D_4_ and C_9_) accompanied by bengamides A and B. Later, from the Indo-Pacific sponge Jaspis digonoxea, Kashman and co-workers [22] identified a new bengazole type product, whose structure was elucidated and named as digonazole (**251**). Not until 1996, Molinski and co-authors [25] identified five new bengazoles (bengazoles C–G, **231**–**235**) from the *Jaspis* sp., comprising a homologous series of *n*, *iso*, and *ante-iso* fatty acid esters (C13–C16) of the same heterocyclic bis-(oxazolyl)methanol parent. Additionaly, Letourneux and co-workers [29] extracted bengazoles from a different sponge, *Pachastrissa* sp. (family Calthropelidae, order Astrophorida), from which they isolated six new bengazole derivatives (**245**–**250**). Some years later, in 2008, bengazoles A, B and E were also obtained from an extract of the sponge *Doryplere splendens* [110]. To date, the bengazole family is comprised by 22 members, isolated all of them from marine sponges [2,22,25,29,109].

The fact that oxazole-containing marine natural products are not frequent in Nature makes bengazoles a rare and intriguing class of natural products characterized by the presence of two oxazole groups linked by a geminal arrangement and a polyhydroxylated appendage [34]. In particular, the 5-substituted and the 2,4-disubstituted oxazoles are biogenetically unusual [111]. The structural difference among the bengazoles A–G lies in the fatty acid moiety linked to the hydroxyl group as ester at the C10 position. Both the relative and absolute configuration of the bengazoles were determined by a combination of NMR studies [34], application of the modified Mosher method [25], analysis of degradation products and chiroptical studies measuring exciton coupling in the CD spectra of the tetra-*p*-bromobenzoate derivatives [25]. Later, total syntheses of some of the members confirmed the assigned absolute configurations.

### 3.2. Biology of Bengazoles

The family of bengazoles exhibits a remarkable ergosterol-dependent *in vitro* antifungal activity against *Candida albicans* [25,29,112,113], displaying comparable values to amphotericin B and suggesting a similar mode of action, as proposed by Molinski and co-workers [23]. In addition, bengazole A (**229**), exhibits anthelminthic activity against *Nippostrongylus braziliensis* at a concentration of 50 µg/mL [34]. Letourneux *et al.* [29] evaluated the antifungal properties against *Candida albicans* CIP 118079, detecting a very active profile for bengazoles **245**–**250**, with MIC values from 0.8 to 1.5 µM. However these compounds did not display inhibitory activity against *Sacharomyces cerevisiae*. On the other hand, Crews and co-workers [109] evaluated their cytotoxicities in the NCI’s 60 cell line screen finding that bengazole A (**229**) exhibited *in vitro* potency against several tumor cell lines (COLO-205 and SK-MEL-5) with GI_50_ values of 0.181 and 1.13 µM, respectively.

### 3.3. Chemical Synthesis of Bengazoles

**Scheme 9:**
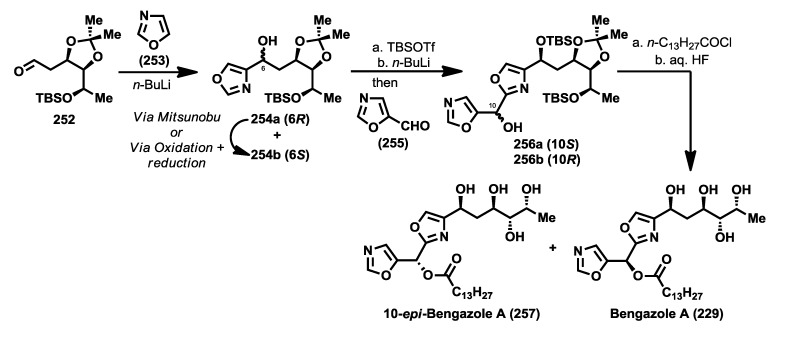
Synthesis of bengazole a and its 10-epimer by Molinski *et al.* [112].

Despite the potent bioactivity displayed by bengazole A, only a couple of stereoselective syntheses have been described. One year after the discovery of the bengazoles, the first total synthesis of one of their members, bengazole A (**229**), was published by Molinski and co-workers [112]. This total synthesis was based on a regioselective metalation/addition strategy. Thus, starting from isoxazole (**253**), and following the addition procedures of lithiooxazoles to aldehydes, established by Hodges and Vedejs [114,115], they reported the synthesis of bengazole A, together with its 10-epimer, in 14 steps with an overall yield of 2.9%. The synthesis started with the addition of 2-lithiooxazole to the aldehyde **252**, readily obtained from d-galactose [116], to yield a mixture of coupled products **254a** and **254b** in a good diastereoselectivity (7:1) in favor of **254a**, justified by a chelation control during the addition process. The stereoselectivity problem in favor of the undesired isomer was overcome by transformation of this isomer (**254a**) into the minor one which contained the right configuration, compound **254b**. This transformation was successfully achieved by subsequent Swern oxidation of **254a** followed by reduction with NaBH_4_of the resulting ketone to provide the correct diasteroisomer for bengazole A. Alternatively, **254a** was similarly converted into **254b** via a Mitsunobu reaction. With sufficient amounts of compound **254b**, a second addition of a lithium species generated by treatment with *n*-BuLi of the silyl ether derivative of **254b** over the isoxazole aldehyde **255**, by modification of Vedejs’ conditions [112,115], furnished an unseparable 1:1 mixture of diastereomers at C10 position **256a** and **256b**. From this diastereomeric mixture, the synthesis was completed by means of a esterification, followed by silyl group deprotection, to deliver an unseparable mixture of bengazole A (**229**) and its C10 epimer **257**, which exhibited comparable antifungal potency compared with pure bengazole A (Scheme 9).

**Scheme 10:**
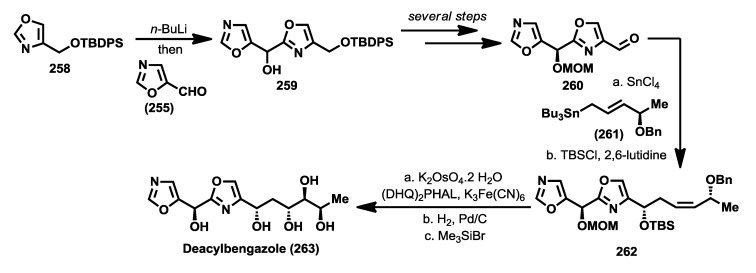
Synthesis of deacylbengazole by Shioiri *et al.* [117].

A second synthesis was reported by Shioiri and coworkers in 2003 in which they prepared deacylbengazole **263** [117]. This synthesis started from 5-formyloxazole (**255**) which was treated with the lithiated derivative of the oxazole derivative **258** to obtain a racemic mixture of the bis-oxazole **259**. Oxidation of **259** with MnO_2_ was followed by an enantioselective reduction of the resulting ketone with the aluminum complex of binaphtol ((*R*)-(+)-BINAL-H) that afforded enantiomerically enriched alcohol in a 78% yield and a 68% of enantiomeric excess(ee) [118]. This compound was then prepared for the introduction of the polyol system by a synthetic sequence that led to aldehyde **260**. For the construction of the polyol system, the 4-benzyloxy-(*E*)-allylstannane **261** was coupled with aldehyde **260** in the presence of SnCl_4_ to obtain compound **262**, after protection of the resulting alcohol as silyl ether. It is important to point out that the coupling reaction between **260** and **261** proceeded in a very high yield (85%) and excellent diastereoselectivity (>99%) in favor of the 1,5-*syn* product. Finally, a Sharpless asymmetric dihydroxylation of the *cis* double bond was accomplished by reaction of **262** with OsO_4_ and (DHQ)_2_PHAL as chiral catalyst to obtain the corresponding diol as a mixture of *syn*/*anti* addition products in 6:7 ratio, which were separated by column chromatography. The correct stereoisomer was then subjected to a sequence of deprotection steps that provided deacylbengazole **263** (Scheme 10).

**Scheme 11:**
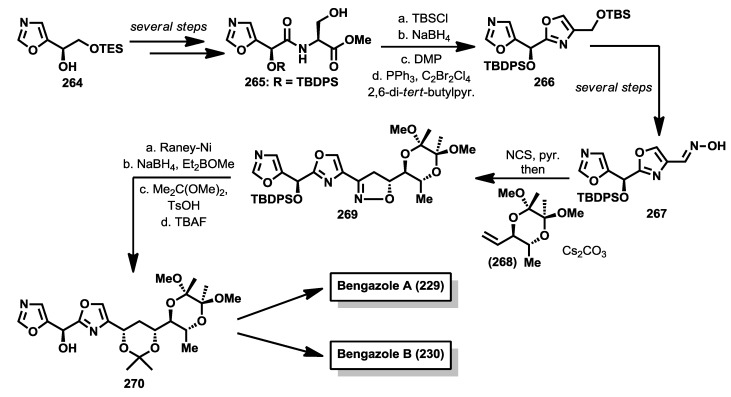
Synthesis of bengazoles A, B and 10-*epi*-A by Ley *et al.* [111].

The following total synthesis of bengazole A was developed in 2006 by Ley *et al.* [111] through a stereocontrolled synthetic route that provided enantiomerically pure bengazole A. This synthesis was based on a convergent strategy featured by the introduction of the C10 steroegenic center earlier than the bisoxazole formation and by the construction of the 2,4-disubstituted oxazole system under mild conditions. As depicted in Scheme 11, the synthesis commenced from the heterocyclic derivative **264**, which was prepared from butane-2,3-diacetal protected glyceraldehyde via a Schöllkopt-type oxazole synthesis [119] by reaction with tosylmethyl isocyanide. Protecting group manipulations of **264**, followed by oxidation using the Jones reagent and an amide coupling with the ester derivative of l-serine afforded amide **265** as a single diastereoisomer. This serine derivative was then easily converted into bis-(oxazol) derivative **266** via a Robinson-Gabriel-type oxazole formation, under conditions reported by Panek and Beresis [120], as a mean of introducing the required second isoxazole ring. At this point, the linkage between the bis-(oxazolyl) containing fragment and the polyol system was programmed via a cycloaddition reaction of a nitrile oxide with an olefin. To this aim, **266** was transformed into oxime **267**, which, by oxidative treatment with NCS yielded the corresponding nitrile oxide that reacted *in situ* with alkene **268** to provide isoxazoline **269** as the major regio- and stereo-isomer. This cycloaddition reaction allowed the construction of the complete carbon skeleton of the bengazoles. To complete the synthesis, the isoxazoline system was reductively cleaved by the action of Raney-Ni, followed by a hydroxyl group-assisted reduction of the resulting β-hydroxy ketone, diol protection and desilylation, to obtain **270**. From this compound, the installation of the myristoyl side chain, followed by the removal of the protecting groups cleavage afforded bengazole A (**229**) with an overall yield of 3.4% over 13 steps. In a similar way, bengazole B (**230**) was efficiently synthesized from the common product **270**. Moreover, the synthetic studies conducted by Ley and coworkers during the synthesis of bengazole A allowed the preparation of the same synthetic intermediates with the opposite configuration at C10 position, thus providing for the synthesis of enantiomerically pure 10-*epi*-bengazole A (**257**) [121].

**Scheme 12:**
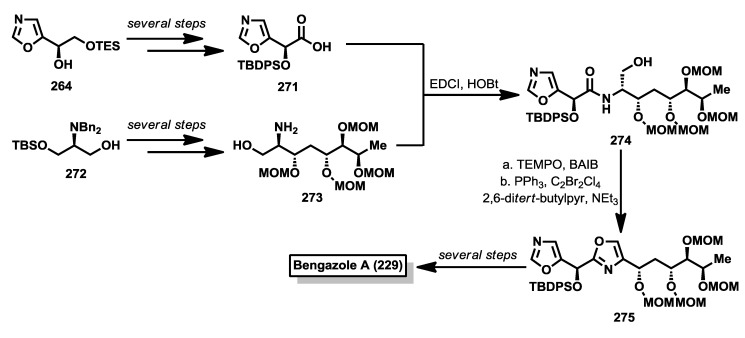
Total synthesis of bengazole A by Chandrasekhar and co-workers [122].

The most recent synthesis of bengazole A was reported by Chandrasekhar and co-workers [122] in 2010. This synthesis commenced from the same synthetic intermediate than the reported by Ley, oxazole **264**, which was prepared by a different synthetic route. This compound was transformed into the desired oxazole acid **271**. Simultaneously, the amino polyol **273** was prepared from the serinol derivative **272** in eight steps, through stereoselective aldol and dihydroxylation reactions [123]. The coupling of both fragments, acid **271** and amine **273**, delivered amide **274** which was converted into the bis-oxazole derivative **275**, according to the same methodology employed by Ley. Finally, the total synthesis of bengazole A was completed by TBAF deprotection of **275**, followed by esterification with myristoyl chloride and final MOM deprotection with TiCl_4_ (Scheme 12). This total synthesis of bengazole A represents the shortest synthesis of this natural product reported to date.

The last contribution to the chemical synthesis of bengazoles was from Gallos and coworkers [124] with the synthesis of the polyol side-chain. This synthetic approach to the bengazoles was carried out by use of d-ribose as starting material for the stereoselective synthesis of the polyol system of the bengazoles.

### 3.4. Design, Synthesis and Biological Evaluation of Bengazole Analogues

**Scheme 13:**
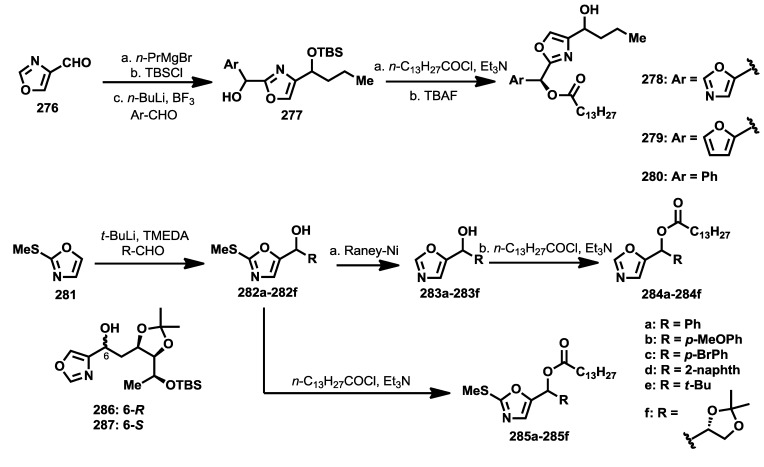
Synthesis of bengazole analogues.

Presently, only analogues of bengazole A have been described in the literature. In particular, in 2009 Molinski and co-workers [113] reported the synthesis and SAR studies of truncated 2,4-disubstituted oxazole analogues of bengazole A and their biological activities against five species of *Candida*. To this aim, derivatives **278**–**280** were prepared from heterocyclic aldehyde **276** by use of different aldehydes (oxazole-5-carboxaldehyde, furfural and benzaldehyde) and generating a small library of analogues that were completely inactive. Undaunted, they decided to synthesize a series of selected 5- and 2,5-substituted oxazoles (**282a**–**f**, **283a**–**f**, **284a**–**f** and **285a**–**f**, respectively) from heterocycle **281** through the synthetic sequence depicted in Scheme 13. To this set of simple analogues, they added compounds **286** and **287**, synthetic intermediates of the synthesis of natural bengazole A, to the list of compounds to be evaluated [112,116]. Unfortunately, biological evaluation of all these compounds resulted in less potent compounds compared to bengazole A and amphotericin B, as detailed in Table 12.

**Table 12:** Activity against *Candida* of selected bengazole analogues (MICs) ^a^.

Compound	R	*C. albicans* ^b^	*C. krusei*	*C. albicans* ^c^
**282c**	*p*-BrPh	4	4	8
**282d**	2-naphth	8	16	8
**283d**	2-naphth	>64	>64	32
**284d**	2-naphth	>64	>64	>64
**285c**	*p*-BrPh	>64	>64	>64
**285d**	2-naphth	>64	>64	>64
Amphotericin B	-	0.50	0.25	0.50

^a^ MIC: Minimum inhibitory concentration (µg/mL); ^b^ ATCC 14503; ^c^ CDFR1, fluconazole-resistant (MIC > 32 µg/mL).

Despite these fruitless results obtained from these analogues in terms of antifungal activity, a correlation can be established related to the presence of a 5-monosubstituted oxazole as well as the fatty acyloxy chain at C10, which potentiates activity only when the polyol chain is linked. On the other hand, these preliminary works on bengazole analogues reflect the need for more elaborated bengazole-type compounds and open new opportunities in the design of novel antifungal agents not yet explored.

## 4. Conclusions

The present review has covered the advances in the synthesis and characterization of the main metabolites extracted from *Jaspis* sponges. Over the period of 30 years since the discovery of the first members of the bengamides and bengazoles, around 50 natural products have been isolated and intense research activity has been carried out in the chemical and biological fields. Numerous authors have described the synthesis of the natural bengamides A, B, E, Z and E′ together with the natural bengazoles A and B, and the procedures designed for their syntheses have improved through the years towards more convergent and flexible processes. In the case of the bengamides, their widely studied cytotoxic profile, has resulted in an intense interest in the synthesis of more potent and efficient analogues. The identification of MetAPs as the molecular targets for the bengamides together with the knowledge of their mode of action has allowed for the rational modification of the bengamide core structure in the design of analogues with improved properties. In such a way, a broad array of analogues (>100) has been generated and biologically evaluated, resulting in a deeper knowledge with regards to their interaction with MetAPs and establishing new insights for the design of more potent analogues with better bioavailability and potential value as new anticancer leads. A prime example is LAF389, which possesses antiproliferative and antiangiogenetic properties as well as antitumor activity. As a consequence of these striking antitumor properties, this compound entered into clinical trials. The chemistry and biology of the bengazoles has, however, been less well explored, due to their biological properties. Additional contributions to the chemistry and biology of these compounds can be expected in the next few years.

In conclusion, although much remains to be done to convert the natural products or their analogues into a drug-like compound, the bengamides in particular are clearly promising targets for future clinical trials and may lead to a better understanding of tumoral diseases and contribute to the development of new leads for cancer chemotherapy.
